# Regulating the Fast-Food Landscape: Canadian News Media Representation of the Healthy Menu Choices Act

**DOI:** 10.3390/ijerph16244939

**Published:** 2019-12-06

**Authors:** Elnaz Moghimi, Mary E Wiktorowicz

**Affiliations:** 1 School of Kinesiology and Health Science, Faculty of Health, York University, Toronto, ON 223, Canada; 2 School of Health Policy and Management, Faculty of Health, York University, Toronto, ON 4700, Canada; mwiktor@yorku.ca

**Keywords:** policy, media, menu labelling, calories, agenda-setting, qualitative research

## Abstract

With the rapid rise of fast food consumption in Canada, Ontario was the first province to legislate menu labelling requirements via the enactment of the Healthy Menu Choice Act (HMCA). As the news media plays a significant role in policy debates and the agenda for policymakers and the public, the purpose of this mixed-methods study was to clarify the manner in which the news media portrayed the strengths and critiques of the Act, and its impact on members of the community, including consumers and stakeholders. Drawing on data from Canadian regional and national news outlets, the major findings highlight that, although the media reported that the HMCA was a positive step forward, this was tempered by critiques concerning the ineffectiveness of using caloric labelling as the sole measure of health, and its predicted low impact on changing consumption patterns on its own. Furthermore, the news media were found to focus accountability for healthier eating choices largely on the individual, with very little consideration of the role of the food industry or the social and structural determinants that affect food choice. A strong conflation of health, weight and calories was apparent, with little acknowledgement of the implications of menu choice for chronic illness. The analysis demonstrates that the complex factors associated with food choice were largely unrecognized by the media, including the limited extent to which social, cultural, political and corporate determinants of unhealthy choices were taken into account as the legislation was developed. Greater recognition of these factors by the media concerning the HMCA may evoke more meaningful and long-term change for health and food choices.

## 1. Introduction

Fast food has a significant presence in Canada. With approximately 10.3 fast food restaurants per 10000 population, more than 54% of Canadians dine out at least one per week [1,2]. The consumption of prepared meals away from home is associated with higher caloric intake and obesity, a factor that contributes to more than half of adult Canadians being deemed as overweight or obese [3,4]. Most consumers who frequent fast food establishments are unaware of the nutritional quality of the menu items, which can vary greatly depending on the restaurant and type of food consumed [5].

With more access to technology, changes in legislation and increasing competition in the marketplace, consumers are starting to become better versed in understanding the ingredients in their foods and are moving towards healthier, more natural and organic food options [6]. In line with this, 27% of Canadians are more likely to visit a restaurant that offers organic or environmentally friendly foods and 57% use nutrition information to make informed decisions, when available [1,7]. It has been proposed that one way to promote healthier food choices is to enhance food labelling to increase consumer understanding, particularly in restaurants [8,9].

One policy designed to promote healthier food choices amongst Canadians is the *Healthy Menu Choices Act* (HMCA). Initially developed as schedule 1 of Bill 45, Making Healthier Choices Act in 2015, the Province of Ontario (Canada) enacted the HMCA, which required restaurants to display caloric information for any food or drink item by January 1, 2017 [10,11]. In enacting the HMCA, Ontario became the first province in the country to legislate menu labelling requirements. The purpose of the Act is to raise public awareness about the caloric content of restaurant foods, enable healthier choices when dining out, and encourage the industry to reformulate high-calorie menu items [12]. However, the results of studies exploring the impact of calorie labelling on promoting healthier food choices are polarized. Several scholarly findings support the legislation. A Canadian study found that menu labelling allowed consumers to use caloric information to make more informed decisions regarding their portion sizes and food choices when dining out [13]. Simplification of food labelling methods to make nutrition information more visible and understandable has been observed to encourage healthier food choices [14]. For example, calorie and nutrient content and energy expenditure information on food labels have been shown to promote healthier food choices among obese individuals [15]. Conversely, other studies have demonstrated that caloric labelling of restaurant and food service establishment menu items was not impactful enough to reduce an individual’s caloric purchasing and consumption [16,17]. Much of this has been ascribed to a multitude of factors, including the food environment, type of restaurant, and cafeteria setting [18]. Overall, the implementation of menu-labelling in real-world settings has rendered mixed results, demonstrating that in some cases it is successful in reducing calories purchased and consumed, and, in other cases, has little effect [19,20].

As part of the Act, business owners who operate in twenty or more locations in the province must display caloric information for their food and drink items and include a context statement under the menus with the recommended daily caloric intake for both children and adults: *Adults and youth (ages 13 and older) need an average of 2000 calories a day, and children (ages 4 to 12) need an average of 1500 calories a day. However, individual needs vary*. Foodservice providers include restaurants that sell both fast-food and cafeteria-style foods, grocery and convenience stores that sell prepared foods, bakeries and coffee shops, movie theatres, and any business that sells ready-made foods for immediate consumption on- or off-site. The caloric content of each food item is posted directly on the menu or, if foods are on display, they must be labelled or tagged with their caloric content. Regulations of the HMCA require that the number of calories in a standard food item be determined by either testing in a laboratory or nutrient analysis method, and the person who owns or operates the regulated food service premise must reasonably believe that the method will accurately estimate the number of calories in the standard food item [11]. Alcoholic beverages are exempt from this law. Public health inspectors monitor businesses to ensure compliance; a failure to comply may result in fines of upwards of $10000 CDN for each day of non-compliance.

Though technical briefing webinars were held for foodservice providers to implement the policy, the dissemination of policies to the general public could be greatly enhanced with the use of mass media, which includes newspapers, television, internet and radio. Given the current accessibility of news media, individuals are more likely to seek health related information from news sources than scientific studies [21,22]. Specifically, use of the internet to obtain health- and science-related news has increased substantially [23,24]. By translating scientific information, news media have become an important means of conveying social norms and hierarchies [21]. The portrayal of health-related policies and shifts in the fast food landscape by the news media may alter societal viewpoints and influence people’s attitudes towards them. For example, news coverage of medical studies has been shown to influence people’s perceptions towards obesity, such that reporting obesity as a public health crisis brought on by bad personal choices can worsen prejudice [25].

Mass media plays an important role in information dissemination, which can impact how these policies are developed and implemented [26]. Public support, or lack thereof, for many policies is strongly associated with their representation in the mass media [26]. Specifically, news media reporting and advertising acts as an agenda setter by revealing (or selectively revealing) information about policies that can shape the perceptions of policymakers and the public [27]. The impact of this media representation is so strong that attention to the policy can rise and fall in response to shifts in media coverage, without any change in the actual size of the problem [28]. In addition, the media greatly influences both policymakers’ perceptions of policy issues and their acceptance by the public [29]. For most of the general public, which includes businesses and households, the main source of information on public policy comes from the media [27]. How the media chooses to gather, analyze and disseminate information on public policies has consequences for how the underlying issues and concepts are socially constructed and the degree of acceptance of public policies [30].

The goal of the current paper was to contribute findings from an analysis of the media representation of the HMCA using mixed methods. Specifically, focus was on how the news media portrayed the strengths and critiques of the Act, which could affect the Act’s success in promoting healthier food choices, and how the Act impacted members of the community, including food service companies.

### Literature Review

News media plays an important role in framing policy debates by setting the agenda for the public and policymakers [31,32,33,34]. How the media represents health policies relies on a network of actors, which include editors, journalists, health professionals, institutions, organizations, policymakers and consumers [35]. Together with the news media, these actors can push their own commercial and political interests and agendas, which can bias the information and message relayed to the general public [36]. The method in which this information is conveyed in the news media can consist of a mixture of emotive and dramatic language that can induce anxiety and fear or highlight significant issues; authoritative sources and statistics that support and give weight to specific claims; calculated placement of powerful actors ahead of less power ones to promote the illusion of a balanced and unbiased story, while giving less value to subsequent speakers; omitting important information that could potentially provide context and another side to the story; and slanting stories in a manner that places blame on specific actors [36]. The social issues journalists decide to report on, and how they do so, can either bring issues to the spotlight or leave them out of public conversations and the consideration of policymakers [37]. For example, an analysis of media discourse on non-communicable disease (NCD) risks and policies found that, while corporations place a greater focus on individual responsibility and draw on market justice frames when discussing NCDs, public health advocates who contribute to news media were more inclined to frame their debates around social justice issues and promoted population-level interventions and structural change [29]. Market justice frames are oriented towards low obligation to the collective good, values of self-determination, and limited government intervention [38].

Similar to the tobacco and alcohol industries, corporations representing fast food tend to have a uniform view on consumption of these products and related policies—which influence arguments made in the press to focus on unhealthy lifestyles, individual responsibility, consumer choice and economic arguments, leading consumption to be seen as a personal choice, an individual level responsibility and a moral issue [29]. Taken together, problematic consumption is typically blamed on the individual having a behavioural problem, rather than framing the issue as a structural societal problem. In this way, attention is diverted from the harmful effects of their own products. These industries can also influence how their products are reported across different types of media. For example, news outlets that relied heavily on advertising income were more likely to have views similar to these corporations [39,40]. In addition, when examining media analyses of processed food and soft drink debates, it was found that they were less likely to expose industry misconduct and its detrimental impact when compared to tobacco-centered debates [29]. This influence can also impact food policies designed to tackle increasing obesity rates. For example, when exploring media representations of Sugar Sweetened Beverage (SSB) taxation as a measure to reduce obesity, industry spokespeople who were interviewed tended to shift the focus away from themselves and focus the debate on individuals’ personal responsibility for their health behavior, saying that their company’s corporate responsibility was ‘part of the solution’ when tackling the obesity epidemic [37,41]. Contrary to public health advocates, who utilized social justice frames to support SSB taxation, the industry was more inclined to draw upon market justice frames that opposed taxation, stating that voluntary action was being taken and that such measures would be anti-competitive [37,41]. With respect to menu labeling laws, data from the US demonstrate that the restaurant industry is strongly opposed, stating that consumers do not feel a need for the information, that the information is readily available, that it is not helpful and is costly for restaurants, and that it represents intrusive government action [42,43]. The market justice frame used aligns with individual blame metaphors and narratives, attributing the cause of obesity to personal choices that call for limited government regulation [44,45].

Media advocacy, which is the strategic use of mass media, including news media, to support and advance healthy public policy, focuses on the ability of the news to mobilize advocates and apply pressure for policy change [46,47]. The use of media advocacy to influence news coverage of policy by public health advocates who seek to highlight the social and political determinants of health and institutional accountability, rather than solely focus on individual responsibility, is therefore important. It is common for journalists committed to covering social issues, such as that of obesity, to produce superficial stories that focus on individual behaviour and treatment rather than social factors that affect prevention [47]. In a study demonstrating how obesity concepts are communicated and propagated in the news media, analysis of 14,302 newspapers that mentioned the word “obesity” determined that the proportion of articles with individual-level framing (27.7–31.0%) was higher than with neutral (18.0–22.1%) or structural-level framing (16.0–16.4%) [48]. This is significant, as numerous studies have demonstrated that the manner in which obesity is framed in newspapers, either through an individual responsibility frame or as a consequence of an “obesogenic” environment, affects the extent to which readers support or oppose policy proposals [49,50,51]. A lack of attention to media framing can give rise to policies such as Commonsense Consumption Acts (CCAs) passed in 25 American states, that shield the food industry from civil liability for claims arising from obesity-related harms [52]. Despite this, there are many public health advocates, coalitions and organizations that support menu labelling laws and other policies that modify the food environment, since they create greater transparency and promote informed decision making [43]. These advocates tend to focus more on the importance of the food environment, using terms such as “toxic” food environment to define the increased availability of junk and fast foods, which are calorie-dense, nutrient-void, and inexpensive [43,51,53]. It is also common for these organizations to use media sources to garner support, gain visibility and influence policymakers [43].

Despite studies of media coverage of American policies, an absence of media analyses of Canadian food regulatory policy prevails. The current analysis sought to address this by clarifying how the HMCA policy was represented in the news media. A focus was placed on the frame used to discuss the policy, how the Act was perceived to impact individuals in the community, corporations and stakeholders, and the extent to which the overall message conveyed in news media considered social, cultural and political influences on food consumption.

## 2. Methods

The data explored in this paper are drawn from a mixed-method study of the representation of the HMCA in Canadian news outlets. An analysis of Canadian national and regional news media, including newspapers, were searched using the electronic databases *LexisNexis, ProQuest*, *Factiva*, and *Google News*. The following search algorithm was used in all the databases:

“healthy menu choices act” OR ((calor* AND label* AND menu) OR (menu-label) OR (menu label)) AND (canada OR ontari*)

Print and digital content published by select Canadian news media, which included newspapers, TV (CBC, TVO, CTV) and newswire (Globe Newswire, UWire) services, were explored. Content published from January 2016 and May 2019 were examined to capture the year that preceded the enactment of the HMCA and the period following implementation. Print and digital news media were collected from both regional and national media outlets to ensure the collection of a diverse sample read by different demographics within the Canadian population. Articles were scanned to ensure they pertained solely to the HMCA and not caloric labelling outside the scope of the legislation.

**Figure 1:**
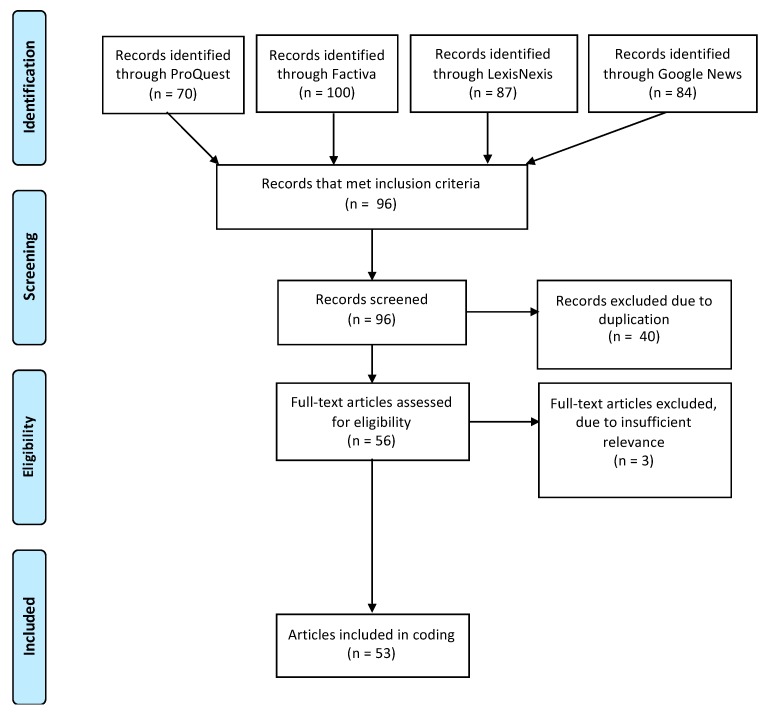
Flowchart outlining the selection of studies included in the analysis.

News articles, opinions/editorials and features were included in the study; however, blogs, press releases, social media correspondence (e.g., twitter feeds), comments from readers, and letters to the editors were excluded. This was to ensure that the written pieces came solely from media outlets rather than members of the general public or the government that developed the Act. Articles were excluded if they: (1) did not acknowledge the Healthy Menu Choices Act; (2) used the Act only as a reference when discussing another topic, such as tobacco cessation; (3) employed the term “calorie labelling” without any reference to the Act or the province of Ontario; and/or (4) were duplicates. Based on these inclusion criteria, 53 articles were selected for analysis. A detailed outline of the search is displayed in Figure 1 and a complete list of the articles and news sources selected can be found in Appendix A.

### 2.1. Quantitative Analysis

A quantitative content analysis was conducted in order to explore the number of publications in national, major city or smaller city news media, the types of articles published and article slant, as well as trends in coverage across time. Articles were categorized according to their slant, using the framework for coding and analyzing media articles on sugar-sweetened beverages [54], childhood obesity topics [55], and smoke-free bylaws [56]. Articles positive towards the HMCA were defined as promoting the act. Articles critical towards the HMCA were defined as outlining the negative repercussions of the act. Articles coded as neutral towards the act solely reported interviews of opinion leaders, research reports, or the status or enactment of the act, with either no opinion or mixed opinions when evaluating the act. News media sources were also categorized as small city, major city and national, to outline the differences in slant across various Canadian demographics.

The analysis was conducted by EM and corroborated through review by MW. To assess inter-coder reliability of the slants, a random selection of 20 percent of articles (*n* = 11) was coded by MW. Coders received 81.8% agreement in this subsample and Cohen’s kappa was a mean score of 𝜅 = 0.672 (95% CI, 0.33 to 1.00), *p <* 0.003, indicating a good level of agreement [57].

### 2.2. Qualitative Analysis

The articles were analyzed using thematic analysis procedures described by Braun and Clarke [58] which determine, examine and analyze patterns (themes) in the data in order to describe a particular phenomenon that pertains to the research questions. Analysis occurred through an iterative process, where news media articles were read several times and coded to determine emerging themes [58,59,60] using NVivo qualitative data analysis software; QSR International Pty Ltd. Version 12, 2018. Codes were developed inductively, and emerging themes were grouped to identify the main themes and subthemes that provided an understanding of the media’s representation of the Healthy Menu Choices Act. Themes explored were related to how the news media represented and portrayed the implications of the enactment of the Act on the fast food industry and the public response to it, including potential benefits and detrimental effects and how the population, stakeholders and government perceived the Act and its effect on them, as well as future policy directions.

The initial analysis and generation of codes was conducted by EM; MW read the articles and supported the refinement of identified themes and sub-themes. Any disagreements were resolved through discussion until consensus was attained. The characterization of themes is supported with reference to quotes within the results.

## 3. Results

### 3.1. Quantitative Findings

Of the 53 articles, 37.7% (*n* = 20) were published in national news media, 24.5% (*n* = 13) in major city news media and 37.7% (*n* = 20) in small city news media. The majority were news reports (71.7%, *n* = 38), followed by opinion pieces (17.0%, *n* = 9) and feature articles (11.3%, *n* = 6). The article publications ranged from February 2016 to June 2018, as illustrated in Figure 3. Two major peaks were observed in the number of articles published: the largest was in January 2017 (*n* = 16), which coincided with the enactment date, followed by a peak in May 2017 (*n* = 11) when a restaurant franchise opposed implementing the policy.

#### Article Slant

**Figure 2:**
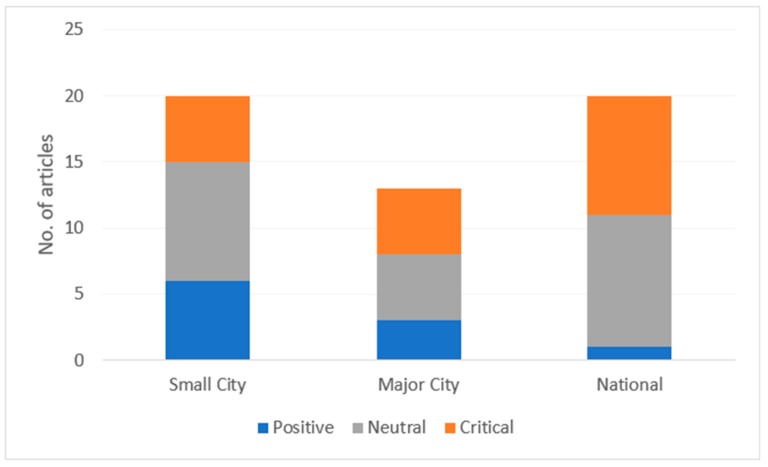
Percentage of articles with a positive, neutral or critical slant, published in small city, major city, and national news media.

Considerable differences were found in article slants published in small city, major city and national news media (Figure 2). In total, 19.0% (*n* = 10) of the articles were positive, 45.2% (*n* = 24) were neutral and 35.8% (*n* = 19) were critical of the HMCA. Major cities had a fairly uniform distribution of positive, neutral and critical articles (*n* = 3, 5, and 5, respectively), while smaller cities mainly published neutral (*n* = 9), followed by positive (*n* = 6) and critical (*n* = 5) leaning articles. National news media articles were nearly equivalent in their neutral and critical slants (*n* = 10 and 9, respectively), while one article reflected a positive perspective.

When assessing article type according to news slant (Table 1), the majority were neutral news reports. In critical slant articles, the difference between the number of published news reports and opinion/editorial pieces was much smaller compared to the other two slants. The majority of opinion pieces were critical of the HMCA. Most of the news reports published reflected a neutral slant. The very few feature articles published consisted equally of either neutral or critical slants.

**Figure 3:**
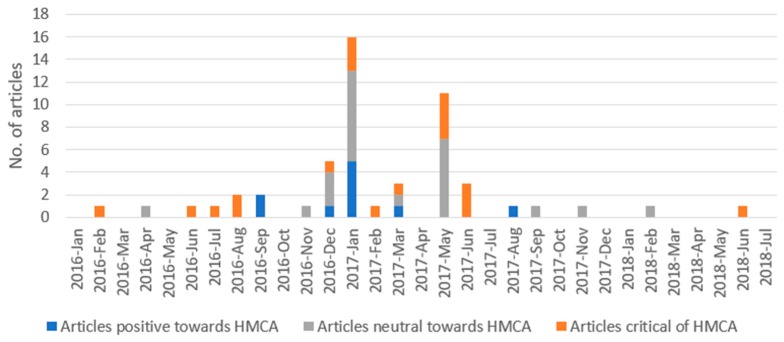
Number of publications and slant over time.

Analysis of article slant over time (Figure 3) indicated that, while neutral and critical articles were spread evenly throughout the study timeframe, the majority of articles with a positive slant were published prior to and inclusive of January 2017, when the act was implemented (*n* = 8).

### 3.2. Qualitative Findings

The thematic analysis reflected a range of areas addressed, presented by incorporating positive, neutral and critical leaning perspectives.

#### 3.2.1. Positive Step Forward

*Informed decisions while dining out:* An important reason why the HMCA was seen as beneficial was due to the high number of Canadians who access meals at restaurants. Indeed, reports stated that, on average, Canadians spend 25% or more of their budget for meals on restaurant foods [61]. Health experts interviewed by media qualified that although the Act will not solve the obesity epidemic, or cause massive change on its own, it is a step in the right direction. The HMCA was described as an important and convenient tool for Ontarians to make informed and healthy decisions regarding their food choices when eating out.

“By having the label readily available, it makes access to this information much easier:

Before they posted these things, you’d have to figure it out yourself. Now you can just look at it and decide quickly.” [62]; Shahid, *Orillia Packet & Times*, 4th January, 2017

Access to the right information was perceived as enabling consumers to be better informed to judge the foods they choose to consume. Most articles quoted health experts when describing the Act’s ability to empower and inform consumers. “You’re able to compare foods and make a more informed decision if you want to try to eat healthier” [63]; Ougler, *Sault Star,* 4th January, 2017.

*Supported by the public, Heart and Stroke Foundation, certain experts*: Reports cited in articles suggested that Ontarians were supportive of the HMCA, despite its impact on franchises [64].

“Who could argue with the policy objective of helping Ontarians make healthier eating choices? No wonder the bill gained the support of both opposition parties.” [65]; Finkelstein, *Financial Post,* 2nd February, 2016

At the same time, the positive impact of the HMCA and its ability to evoke healthier change was typically reported the most in smaller city news media: “Diners are reacting positively to new regulations that require some Ontario eateries to provide information about the calorie counts for dishes, says one Durham restaurateur” [66]; Mitchell, *Oshawa This Week,* 2017.

In addition to government and public support, several articles stressed that the Heart and Stroke Foundation of Canada, a cardiac health-based foundation, praised and viewed the initiative “…as an important tool in our toolbox providing us with information that will help us make better choices” [67]; Laws, *Belleville Intelligencer* 3rd February, 2017. Only one article mentioned the support of The Fitness Industry Council of Canada [68]; Brynna, *Ottawa Citizen,* 15th April, 2016, while multiple sources cited such health experts as physicians, dieticians, researchers and advocates as supportive of the Act. Although such health experts believed its passage was a positive move that other provinces would emulate, their comments were almost always accompanied by critiques that the Act was insufficient to drive mass change on its own:

“The likelihood of this single change having a dramatic impact on diet-related chronic disease incidence in Canada is low, but that doesn’t mean it’s not an important thing to do.” [69]; Foote, *CBC News,* 3rd January, 2017

#### 3.2.2. Will Evoke Future Change

*Additional food labelling*: Health experts cited in articles stated that the next step in food labelling should include nutrient content, with a significant focus on sodium. “…diners ought to be given even more information about their menu choices…in the future, information on fat and sodium levels may be of value for health-conscious customers” [70]; Crawford, *Ottawa Citizen,* 3rd January, 2017.

*Greater transparency in food and menu formulations*: One of the most serious problems health experts interviewed emphasized was the large portion sizes at restaurants that drive weight gain and unhealthy food choices; they suggested that a potential benefit of the HMCA was that it would encourage consumers to opt for smaller portion sizes. “…posting calorie counts will dissuade a lot of people from ordering massive plates of food, or those sneaky calorie-packed breakfast sandwiches, specialty coffees and soft drinks” [68]; Brynna, *Ottawa Citizen,* 15th April, 2016. By providing caloric numbers, health experts in turn predicted that companies would be forced to become more transparent in how they formulate their products and the ingredients they would choose to include in them.

Specifically, articles stated that the HMCA would encourage restaurants to reformulate their products to include healthier, low-calorie options. Some experts explored potential “indirect effects” of menu labeling laws, including ‘social spillover.’ In an effort to attract health conscious consumers, food outlets may launch new lower calorie options to address public demand [71]; Hauser, *Sault Ste. Marie This Wee*k, 2017.

*Evolution of lifestyle trend*: One media report suggested the Act could reinforce a trend in preference for healthier food consumption in the same vein as the rise of organic and locally produced food choices that will only advance in the future. The article suggested that society is poised for a food revolution, similar to the normalization of seatbelts, which the car industry resisted before realizing the business opportunity.

“Nowadays, car manufacturers spend billions of dollars developing advanced safety features in an effort to stay competitive in an increasingly protection-minded marketplace. The seatbelt comparison…remind(s) us that this isn’t the first time…policy makers have had an uphill battle: educating consumers who might prefer to keep their heads in the sand while prodding a recalcitrant industry to take some responsibility” [71]; Hauser, *Sault Ste. Marie This Week*, 2017.

Canadian legislation that phased out the use of trans fats (largely from partially hydrogenated oils) reflects another recent example of this trend [72].

Although many articles praised the importance of menu labelling as evoking future change, one article emphasized that the positive change will be reinforced at the federal level, with the potential to extend nationwide:

“…the Canadian Food Inspection Agency is in the midst of a food labeling modernization initiative, suggesting that menu labelling and food labelling reform will remain an important issue in the years to come” [73]; Mayzel and Leszcz, *RestoBiz,* 19th September, 2017.

#### 3.2.3. Association with Health Outcomes

Although the intent of the legislation was portrayed as encouraging Ontarians to make healthier decisions, there was ambiguity on the definition of “health.” Many articles created a causal definition of health by conflating it with weight. “Given that 40+ per cent of Canadians today classify themselves as overweight or obese, the tracking and display of caloric information is a positive step in the right direction” [74]; Couto, *Toronto Sun,* 13th May, 2017. Weight was thus characterized as a typical measure of health, with the premise that the Act centers on addressing obesity and little acknowledgement of other NCDs associated with unhealthy dietary habits. The very few articles that mentioned illnesses associated with unhealthy eating patterns were also linked to weight. “The longer they maintain unhealthy weights, the more vulnerable they are to the early onset of debilitating conditions such as heart disease, diabetes, cancer and joint damage” [75]; Sonnenberg, *Simcoe Reformer,* 5th January, 2017.

#### 3.2.4. Cost Implications for Restaurants

Due to the high cost of developing and implementing menus with caloric labelling, many small businesses were vocal about the financial strain associated with the HMCA. Restaurant owners were concerned about the cost and complexity of the new menus. “It’s a lot more complex than it looks…there are 10000 different ways to make a sub” [76]; Warhaft-Nadler, *Huffington Post Canada,* 23rd May, 2017. Given these complexities, some restaurants were reported to disregard incorporating healthier options, which was counterproductive to the intent of the Act.

Large franchises and fast-food restaurants were found to experience fewer difficulties than their smaller counterparts; articles reported that major chains plan to be compliant [77]; Wright, *Toronto Star,* 9th July, 2016. Several articles reported the Ministry of Health hired consultants (Fleishman Hillard) who are a known lobbyist for the fast-food industry to gather public consultations on menu labelling and to develop a report on the issue [77]; Wright, *Toronto Star,* 9th July, 2016, which was considered a conflict of interest.

“’They have a clear conflict of interest there because they’re working for companies that are creating a lot of the problem,’ said Bill Jeffery, executive director of the non-profit Centre for Health Science and Law.” [78]; Leslie, *CTV News,* 11th August, 2016.

The same health expert interviewed suggested that the reason for working with the agency was to gain favours from large corporations. The immense challenge for health agencies and policymakers to educate consumers resistant to change and incentivize a reluctant industry to take a level of responsibility was stressed in another article [71]; Hauser, *Sault Ste. Marie This Wee*k, 31st August, 2017.

#### 3.2.5. Questionable Benefits

*Focus on calories* vs. *nutrients:* While many articles discussed the importance of nutrient labelling, many held that healthy eating is more complex than counting calories, a concept seen as missing in the HMCA [79]; Wright, *Toronto Star,* 14th September, 2016. Concern about the potential of the HMCA to effectively erode the greater nutritional context of a meal was thus expressed “…(O)ur tendency to view food as a cumulative number has stripped nutrition of valuable nuance and contributed to a false sense of dietary understanding” [80]; McNeilly, *National Post* 06/05/2017. The same article suggested the HMCA could be misused by companies to legitimize processed foods by presenting them as health-inclined, for example by enabling “…soda companies to proudly boast that cans of soda only have 120 calories, or that candy bars only have 210 calories, all while ignoring what the high level of calories from sugar do to the body’s metabolic function” [80]; McNeilly, *National Post* 5th June, 2017. Two articles described the negative impact this would have on children [76,81]; Warhaft-Nadler, *Huffington Post Canada,* 23rd May, 2017; Luciani, *Hamilton Spectator,* 27th June, 2017, who would be more susceptible to viewing calories as the only measure of health. “Kids are afraid of calories and fat, and will lean towards foods with less calories even if they also have less nutritional value, simply because we are a society that is completely fat phobic” [76]; Warhaft-Nadler, *Huffington Post Canada,* 23rd May, 2017.

A publication peak occurred after a restaurant (Freshii) marketing itself as “healthy” fast food initially announced it would not follow the HMCA [82]; Birak, *CBC News*, 8th May, 2017. Despite the restaurant indicating that calorie labels are simplistic, mislead their guests and distract from their focus on nutrients, they decided to comply to avoid fines and instead invest in menu innovation and partnerships that advance health in communities [83]; The Canadian Press, *Globe Newswire,* 9th May, 2017.

The long-term benefits of the policy and its ability to evoke a meaningful change was questioned in multiple reports and health experts believed that, in order to address the underlying issues, focusing on and oversimplifying one facet is insufficient to evoke change, especially for diet-related conditions like Type 2 diabetes, obesity, hypertension, and others, whose causes are more complex, and a myriad of interventions designed to address them are needed.

“Framing the question as, ‘Will this or will this not help with obesity?’ is an unfair way to categorize it. It’s like asking, is that particular sandbag going to stop the flood when what you really need is a whole slew of sandbags.” [70]; Crawford, *Ottawa Citizen,* 3rd January, 2017

*Inaccuracies in the context statement:* The legitimacy of the initial recommended caloric intake for women in the context statement, which must accompany the calorie information in chain restaurants, was questioned in two articles [84,85]; Mintz, *TVO,* 16th January, 2017; Wright, *CBC News,* 10th August, 2016. The statement indicated that adults require 2000 to 2400 calories per day, but that individual calorie needs vary. Bill Jeffery, executive director of the Centre for Health Science and Law, cited in one article, specified that the number is far too high, especially for women, referring to the Health Canada recommendation that women aged 31–50 with a low level of activity consume 1800 calories a day [77]; Wright, *Toronto Star,* 9th July, 2016. An Ontario Ministry of Health spokesperson noted that the context statement was being reviewed in light of the comments. In 2018, the Act was amended to remove the statement that included the range of calories (2000–2400). Instead, only the first statement, that adults require an average of 2000 calories, is to be included, suggesting the expert critique may have influenced the amendment to the context statement.

*Weak scientific evidence to support menu labelling:* Numerous articles cited scientific evidence of the failure of similar policies in other jurisdictions to change eating behaviour and nutritional intake. They emphasized the Ontario government neglected American research on the impact of labelling laws in operation since the early 1990s, that found that these policies were ineffective in prompting healthier food choice, weight loss or reduced obesity levels [81]; Luciani, *Hamilton Spectator,* 27th June, 2017. A study cited in one article that monitored consumption in a Starbucks in New York found that posting calorie content led to a six per cent reduction in calories consumed by customers that lasted for at least ten months after implementation, with revenues unaffected [86]; Ward, *UWire,* 10th January, 2017.

Despite compelling research concerning the weak scientific evidence forming the basis of the HMCA that media reports stressed, an article highlighted the government’s unwavering support for the Act as part of the solution to childhood obesity, and similar legislation enacted by other international cities [87]; Warren, *Metro News,* 5th January, 2017.

*Unlikely to alter food choices:* One reason that individuals may not make positive use of the HMCA mentioned, is the limited awareness and understanding of the significance of calories and how to use the information [67]; Laws, *Belleville Intelligencer* 3rd February, 2017. Many articles stated that the HMCA was not strong enough to evoke a meaningful change. They noted that when people frequent fast food establishments, they already know what they want and that menu labelling is unlikely to deter their decision; moreover, “…nothing will change that other than higher taxes.” [81]; Luciani, *Hamilton Spectator* 27th June, 2017. Another article also suggested taxing junk foods to pay for the cost of healthcare, an approach similar to that used in the tobacco epidemic [67]; Laws, *Belleville Intelligencer* 3rd February, 2017. The tobacco analogy was also used to illustrate people’s relationship to fast foods:

“There are still people who buy cigarettes even though the package clearly states that using this product will kill you. If lattes are your vice, that little number on the menu isn’t likely to scare you away from a pumpkin spice.” [88]; Brown, *Waterloo Region Record,* 7th January, 2017.

A small number of articles acknowledged that, although most people will not change their food options, the transparency that labelling enables might make them opt for healthier choices the next time they visit the establishment. As a result, multiple restaurant owners and managers interviewed in articles stated that they did not see a significant difference in the food choices of their customers, with one corporate chef stating, “this is the place you go to cheat” [77]; Wright, *Toronto Star,* 9th July, 2016.

#### 3.2.6. Impact on Marginalized Populations

*Eating disorders:* Those suffering from eating disorders such as anorexia and bulimia nervosa were considered to be adversely affected as a consequence of the HMCA. “People recovering from eating disorders will find it harder to stop constantly calculating their food intake” [89]; Robertson, *Metro News,* 4th January, 2017. One health expert cited in numerous articles petitioned to remove menu labelling due to the dangers it posed for those suffering from eating disorders. An article also pointed out that, although a spokesperson for the Ontario Ministry of Health stated that the government spoke to restaurants, health professionals, dietitians, and groups exploring the impact on eating disorders, he did not specifically refer to any eating disorder advocacy groups or individuals with lived experiences with whom they consulted [90]; Robin, *TVO,* 2nd June, 2017.

*Women:* As women are more likely to read and use nutrition labels compared to men, the inaccurate context statement could lead to weight gain in this group:

“Recommending up to an extra 600 calories a day for women could make a huge difference. Even an extra 200 calories per day can contribute to unhealthy weight gain.” [77]; Wright, *Toronto Star,* 9th July, 2016.

*Schools*: One place exempted from the HMCA was schools; an article emphasized that schools are to comply with different legislation that requires them to offer a healthy choice [91]; Petrick, *Belleville News,* 1st March, 2017. However, several articles were vocal about this decision not being appropriate for school aged children:

“Those exemptions prompted the board to agree to send a letter of complaint to the Ontario Ministry of Health and Long-Term Care…young people need to learn to make healthy food choices and ‘it’s critically important’ such information be available to them.” [92]; Hendry, *Trenton Trentonian,* 9th March, 2017

*Low income:* One article highlighted that the degree to which food labels are used and healthy eating behaviours followed differs between low and high income individuals, as low income consumers are more likely to ignore labeling than those with a high income. “The very people whose behaviour the legislation is geared to influence seem immune to the labelling laws, making calorie counting a waste of time and money” [81]; Luciani, *Hamilton Spectator* 27th June, 2017.

An alternate suggestion to more meaningfully approach the reduction of obesity and associated rise in health care costs was to address high food prices, to encourage Canadians to eat healthy, nutritious food, at home.

“…(B)uying fresh fruit and vegetables, as well as meat for recipes…takes up a very large portion of any grocery budget and I really only have to worry about myself, I can’t begin to imagine the costs for a family of four with growing children.” [67]; Laws, *Belleville Intelligencer* 3rd February, 2017.

## 4. Discussion

In reporting on both the benefits and critiques of the HMCA, many Canadian news media considered the Act a first step in the right direction for consumers to make informed decisions concerning healthier choices, an important factor considering the large number of Canadians who access meals away from home. Though most health experts supported the HMCA, they questioned its benefits and ability to drive mass change on its own. In terms of slant, most articles were neutral, followed by critical, and, lastly, positive. Opinion pieces were largely critical of the HMCA. Small city news media published the majority of the positive slant articles. Among the news media, health was strongly conflated with weight and high calorie consumption, with little acknowledgement of the link between unhealthy eating behaviours and other illnesses.

Accountability for healthier eating choices was often placed on the individual, with little consideration of the structural factors that affect individual choice, including the role of the food industry and density of fast food outlets. A study of the United Kingdom (UK) Household Longitudinal Survey, for example, found that populations most exposed to away-from-home food establishments had 16% greater odds of allocating more than 25% of their food expenditures to them [93]. In the United States, low-income populations were more likely to live in communities that did not support healthy choices [94]. Although both the benefits and critiques of the HMCA were conveyed, news media focused more on changing the food choices of consumers, rather than addressing the broader structural issues, such as the food industry and government social and economic policies that play a significant role.

The positive implications of the HMCA as an emerging trend were most reported in small city news media, compared to large city and national sources. As the main driving factor for global obesity rates is the rise in BMI in rural regions compared to more urban areas [95], the greater prevalence of positively slanted articles in small cities was found to parallel the concern for rising obesity rates in those regions.

Positive reports cast the Act as encouraging consumers to make informed decisions, despite the likelihood of being insufficient to evoke meaningful change on its own. A recent meta-analysis demonstrated that restaurant menu labeling did not significantly impact total calorie intake or alter the nutrient profile of consumer food choices [96]. Opinion pieces written by health advocates were more likely to stress the importance of the nutrient rather than the caloric content of food as an important factor in determining the “healthiness” of a food. This aligned with the views of health experts at a consensus conference to determine the best mode of action on nutrition labelling in Canada—that the availability of ingredient and nutritional information from restaurants would be beneficial [97]. Caloric labelling alone is therefore insufficient, as factors such as culture, awareness, attitude, and structural aspects such as access to healthy and fast food venues, as well as financial incentives, contribute to the change sought [98,99]. A controlled restaurant field experiment found that, while calorie labeling reduced the number of calories ordered by health value-oriented consumers, in contrast, it may increase calories consumed by consumers who are quantity-value and taste value-oriented [100]. Low socio-economic groups are more likely to be represented among consumers with these hallmarks [100].

Given that the HMCA was not viewed as sufficiently impactful to change the dietary habits of individuals, media representatives of fast food giants did not negate or criticize the policy in news media articles. Most fast food giants reported in the news (e.g., Starbucks, Harvey’s, McDonald’s), were on board with the HMCA compared to their smaller sized counterparts, who found menu labelling to be costly. A feasibility study exploring the ability of independent restaurants in Ontario to implement menu labelling demonstrated that it is only feasible for restaurants that are motivated and ready to commit, offer healthy foods, have standardized recipes and receive support, including recognition and cost offsetting [101]. Smaller restaurants may lack some of these features, particularly when it comes to support and cost offsetting, which may be why they are less inclined to promote the Act. Food labeling has also been shown not to significantly impact industry product formulations for total energy [98], despite their being notorious for high calories and limited nutrients essential to good health. It is possible that the focus of the Act on calorie rather than nutrient content reflects the control and influence of these companies on public policy. Important examples of this were in the articles that described how representatives of fast food companies worked with policy makers to develop the context statement. Despite the conflict of interest these articles reported, they emphasized the power of the food industry to influence dietary recommendations [102].

Taken together, media reports vested much of the accountability for food choices in the hands of the consumer, with little acknowledgement of the broader factors that impact individual decisions. Very few articles acknowledged the role that the food industry has in promoting unhealthy food choices and eating behaviours. The subtle tone of the news articles aligned strongly with a personal responsibility frame, which can give rise to “healthism” by conveying the point that “healthy” and “responsible” people will make good food choices if they are given the information, and that the entire issue of hyperpalatable foods should be reduced to an individual’s sense of moral responsibility, with little to no acknowledgement of the broader structures and forces that make it difficult for people to access healthier food options. This also aligns with reports in the literature, which state that 72% to 98% of obesity-related news reports emphasize individual responsibility for weight [103]. To advance a better informed public dialogue concerning feasible and pragmatic solutions to public health dilemmas, it is important for the news media to access research sources that enhance their understanding of how issues may be framed beyond a superficial perspective, to consider broader social, cultural and political influences on food consumption and shared accountability [47]. Opinion pieces were more likely to explore in-depth influences compared to news reports.

Within the articles, an overt focus on weight and calories linked to health prevailed, that frequently sensationalized weight gain by using the terms “unhealthy weight gain”, “obesity” and “obesity epidemic” when describing the consequences of high caloric consumption. Urging consumers to make better dietary choices for the single purpose of losing weight can be categorized as a “fat frame” that can shape a culture’s orientation towards the meaning of fatness [25]. In communication, frames display how news media constructs particular accounts of social problems, which can impact which solutions appear legitimate and feasible [25,33]. The frame of defining health as weight was largely endorsed, and very few journalists discussed the impact of the policy on other non-communicable diseases in the Canadian population. The weight-based stigma and prejudice that may arise with this frame of defining health may prove to be counterproductive and create stress, poor health and barriers to healthcare [104,105].

The repercussions of this “fat-phobia” can fuel incorrect notions of what health is and exacerbate the condition of those with pre-existing eating disorders. Articles referenced cases where individuals diagnosed with eating disorders like anorexia nervosa or bulimia nervosa would view the labels as triggers that could lead them to fall back into patterns of caloric restriction or purging. The media has a strong role in perpetuating the idea that women are preoccupied with their shape and weight [106,107] and pay more attention to menu labels. When compared to men, studies have found that women were more likely to use menu labels and consume less calories [16,17,108,109]. Hence, the use of caloric labeling on menus as a trigger for eating disorders centered largely around females, with several references to a researcher who petitioned against the legislation. Many articles raised this issue as a reason not to pass the Act. It would be important for future studies to examine whether the news media has a role in influencing these behaviours amongst women.

An important limitation of the study was the small number of articles identified and included. Only 53 unique articles were published on this topic, far less when compared to the portrayal of other policies in the media. For example, studies analyzing the UK media portrayal of the taxation of sugar-sweetened beverages within a similar timeframe identified 511 and 374 articles, respectively [54,110]. There are several reasons why this may have occurred. Importantly, because the law was only passed in Ontario, its lack of impact at the national level may have deterred news sources from reporting extensively on the topic. In addition, many of the reports had redundancies; as novel insights were not added, reporting may have diminished post-enactment. Only when a restaurant was against implementing the Act was there a sharp spike in reports, which declined once the restaurant complied. Though the media is an important tool in initiating policy discussions [111], the low number of reports on this topic may have been due to factors such as low demand and interest in the topic, and preference to simply report the existence of the Act, rather than initiate meaningful dialogue that could change the impact of the food industry on how the issue was framed, or influence the policy.

### 4.1. Policy Implications

Simplification of food labelling to make nutrition information more visible and understandable is one approach to promote healthier food choices [14]. As the current study found, policies based on outdated conceptualizations, or integrated into a community whose understanding of how to use the information is limited, may have a narrow policy impact. Research concerning the effectiveness of menu labelling as a sole initiative is sparse and weak. Similar policies implemented in other regions were found unsuccessful in changing eating patterns, food choices and obesity rates [18,112,113]. Focusing on a policy with limited effectiveness in an unchanged obesogenic environment may shift attention away from fostering healthier food and eating environments [114]. It is important for future studies to explore the media’s promotion of unsuccessful food policies and whether this creates a discrepancy between perceived and actual healthy eating environment. Exploring how these inferential errors and misrepresentations impact NCD and obesity rates could lend further insight [115,116].

Since the media can reflect the community’s reception to the Act, superficial reports reflect a missed opportunity to educate the public and inform government and industry policy. Many media reports failed to align with research results that clarify that myriad social, cultural, political, health, and corporate factors must be considered to ensure policy success. Some of these concerns were conveyed in the news media, despite stating that the HMCA may be a positive first step. Research supports the need for a more nuanced understanding of the regulation of consumption patterns, that considers personal, genetic, environmental and social structural factors that affect health status [21,103,117]. Adding these factors as educative components may allow individuals to better understand how to use caloric labelling and enhance the potential to make healthier food choices. Educational support in tandem with anti-smoking regulations have, for example, had a positive impact on tobacco cessation [118,119,120]; a multi-pronged approach is more likely to be effective as nutritional policy. Taken together, although the media highlighted the insufficiency of making the public aware of the caloric content of their food to realize the policy goal, they failed to emphasize the more important issue of addressing the structural determinants of healthy food consumption as part of a policy intended to evoke meaningful change in food choices and eating habits.

### 4.2. Conclusions

Although most news articles considered the introduction of the Healthy Menu Choices Act as a good start to inform consumer decisions, they highlighted the criticism of health advocates concerning the ability of the Act to evoke long-term and meaningful change. Corresponding to findings in the literature, media portrayal of the HMCA perpetuated an individual responsibility frame that failed to recognize the contribution of fast food restaurants and the social structural determinants to healthy menu choices. Most notably, the simplistic conflation of health and weight led to the characterization of health as solely impacted by calorie content. Although experts interviewed in news articles attributed the outdated model on which the HMCA is based and the potential conflict of interest to its failure to acknowledge the nutrient content of food, a personal responsibility frame, focused largely on changing the behaviour of individuals in fast food restaurants, was generally maintained. A more balanced frame could have also addressed what these establishments can do to promote healthy eating behaviours, and how government policy could better regulate environmental, nutritional access and social structural aspects. A more in-depth analysis by the media could illuminate the potential for the government to establish stronger incentives and regulations for the food industry, and enhance societal understanding of nutrition, so that community members may better use the information to make informed decisions. It is important for the media to recognize the contributions of social, cultural, political, health and corporate factors that influence community response to the HMCA, and offer a more realistic depiction of the Act to the public.

## Figures and Tables

**Table 1:** Article types categorized by article slant.

	Article Type		
Slant	News Report (%, *n*)	Feature Article (%, *n*)	Opinion/Editorial (%, *n*)
**Positive**	21.1%, 8	-	22.2%, 2
**Neutral**	52.6%, 20	50%, 3	11.1%, 1
**Critical**	26.3%, 10	50%, 3	66.7%, 6

## Appendix A

**Author**	**News Outlet**	**Title**	**Date Published (yyyy/mm/dd)**
Finkelstein, C	Financial Post	Bafflement behind a policy meant to help Ontarians make healthier eating choices	2016/02/03
Brynna, L	Ottawa Citizen	Calorie counts will make consumers think twice when eating out	2016/04/15
Melnitzer, J	National Post	'Joint-employer' status could rattle industry; Franchising based on brand uniformity	2016/06/15
Wright, Lisa	Toronto Star	Calorie counts on menus a costly headache and source of confusion for eateries	2016/07/09
Wright, Lisa	CBC News	Ontario fast-food labels could cause women to gain weight, publish health advocate says	2016/08/10
Keith, L	Telegraph Journal	Ontario bowed to food industry pressure on menu-labelling legislation, critics say	2016/08/11
Wright, Lisa	Toronto Star	Latte or lemon loaf? Calorie counts coming to Starbucks	2016/09/14
Nguyen, L	Vancouver Sun	Starbucks to post calorie counts at Canadian locations	2016/09/15
Finkelstein, C	Financial Post	New regulations on the books that could affect your franchise	2016/11/16
	CBC News	Food menu calorie counts dangerous for people with eating disorders, experts warn	2016/12/29
	CBC News	How many calories in that fast-food meal? Ontario menu labelling legislation takes effect Jan. 1	2016/12/29
	CTV Ottawa	Calories coming to menus in January	2016/12/30
	Ottawa Citizen	Calorie count rollout for diners	2016/12/31
Cross, B	Windsor Star	Menu items must include calorie count as of Jan. 1; Restaurants with fewer than 20 locations exempt from new law	2016/12/31
Weidner, J	Waterloo Region Record	Calorie counts on menu dangerous for people with eating disorders: advocate	2017/01/01
Crawford, B	Ottawa Citizen	Will Ontario’s new calorie labelling law affect what people eat?	2017/01/03
Foote, A	CBC News	Calories on menus ‘one sandbag in the levee’ against weight problems	2017/01/03
Ougler, J	Sault Star	Healthy helping for diners; Dietician says putting caloric information on restaurant food and drinks a great idea	2017/01/04
Robertson, DC	Metro News	Ottawa dietician weighs in on menu calorie counts	2017/01/04
Shahid, M	Orillia Packet & Times	Calorie counting now on the menu	2017/01/04
Sonnenberg, M	Simcoe Reformer	New tactic deployed in battle against obesity	2017/01/05
Warren, M	Metro News	Calorie counts on menus worry Toronto eating disorder advocates	2017/01/05
Brown, C	Waterloo Region Record	Every tasty fry, every pizza pie, I'll be counting you	2017/01/07
Kula, T	Sarnia Observer	Calorie counts a good start, nutritionist says; Calorie listings on many menus in Ontario started Jan. 1	2017/01/07
Cudworth, L	Stratford Beacon Herald	Calorie information is 'just one tool'; New provincial legislation requires food chains to list calorie counts	2017/01/09
Ward, J	UWire	The Ontario government keeping track of calorie consumption	2017/01/10
Mintz, C	TVO	Ontarians are finally learning to count their calories	2017/01/16
Mitchell, J	Oshawa This Week	Durham diners eating up calorie info required by new law	2017/01/24
Ross & McBride LLP	Hamilton Spectator	Legal Matters: Understanding the healthy menu choices act	2017/01/24
Baranyai, R	Simcoe Reformer	Caloric counts on menus may not make difference	2017/01/28
Laws, J	Belleville Intelligencer	Calorie legislation seems to be too little, too late	2017/02/03
Petrick, S	Belleville News	Know your calories before ordering – it’s the law	2017/03/01
Hendry, L	Trenton Trentonian	Strengthen menu rules: board	2017/03/09
William E & Osinga N	Oshawa This Week	Lakeridge Health dietitians weigh in on calorie counts	2017/03/22
Birak, C	CBC News	Freshii food chain a holdout in posting calories on menu boards	2017/05/08
Goffin, P & Sagan, A	Toronto Star	Freshii calls Ontario calorie-counting law ‘overly simplistic’	2017/05/09
Sagan, A	CTV News	Ontario government says Freshii not posting calorie info on menus	2017/05/09
The Canadian Press	Toronto Sun	Freshii in hot water for not posting calorie info on all menus	2017/05/09
The Canadian Press	Globe Newswire	Count Nutrients, Not Calories	2017/05/09
McNeil, S	BNN Bloomberg	'We're not going to be martyrs': Freshii complies with calorie-count legislation	2017/05/10
Sagan, A	Chronicle Herald	Ont. says Freshii not posting calorie information on menus	2017/05/10
Sagan, A	The Globe and Mail	Freshii calls Ontario calorie count rules ‘simplistic’ in wake of inspection	2017/05/10
The Canadian Press	National Post	Ontario Puts Heat On Freshii For Calorie Counts	2017/05/10
Couto, S	Toronto Sun	Calories Vs Nutrition	2017/05/13
Warhaft-Nadler, M	Huffington Post Canada	Calories On Menus Are Making Healthy Choices Even Harder	2017/05/23
McNeilly, C	National Post	The salad or the Big Mac? Why the calorie in, calorie out argument keeps failing us	2017/06/05
Robin, I	TVO	How calorie counts on restaurant menus harm those with eating disorders	2017/06/12
Luciani, P	Hamilton Spectator	Counting calories is bad science and doesn't work anyway	2017/06/27
Hauser, M	Sault Ste. Marie This Week	Something for Ontarians to chew on	2017/08/31
Mayzel E & Leszcz N	Restobiz	Ontario’s menu labelling law check-up	2017/09/19
Ogilvie, M	Toronto Star	Is this noodle soup too massive for lunch?	2017/11/15
	Chronicle Herald	Pita Pit Canada rolls out full-calorie info	2018/02/13
Hildebrandt, S	Toronto Star	Link found between eating disorders and gender identity	2018/06/29
